# Medication adherence in HIV-positive patients with diabetes or hypertension: a focus group study

**DOI:** 10.1186/1472-6963-13-488

**Published:** 2013-11-25

**Authors:** Anne K Monroe, Tashi L Rowe, Richard D Moore, Geetanjali Chander

**Affiliations:** 1 Department of Medicine, The Johns Hopkins University School of Medicine, Baltimore, Maryland, USA; 2 Department of Pediatrics, The Johns Hopkins University School of Medicine, Baltimore, Maryland, USA; 3 Division of General Internal Medicine, 1830 E Monument St, Room 8060, 21287, Baltimore, MD, USA; 4 Division of General Pediatrics and Adolescent Medicine, Research Program Coordinator, 200 N. Wolfe St, Suite 2096, 21287, Baltimore, MD, USA; 5 Division of General Internal Medicine, 1830 E Monument St, Room 8059, Baltimore 21287 MD, USA; 6 Division of General Internal Medicine, 1830 E Monument St, Room 8047A, Baltimore, MD 21287, USA

**Keywords:** Diabetes, Hypertension, HIV, Adherence

## Abstract

**Background:**

People with HIV are living longer with potent antiretroviral therapy (ART), and HIV is increasingly complicated by other chronic medical comorbidities. The objective of this study was to explore HIV-positive patients’ perspectives on living with HIV and diabetes mellitus (DM) or hypertension (HTN) and factors affecting medication adherence.

**Methods:**

We conducted six focus groups. Two investigators independently coded transcripts for thematic content using editing style analysis. Codes were grouped into conceptual themes using consensus process.

**Results:**

Thirty-five HIV-positive patients with diabetes or hypertension participated. Four major themes emerged: (1) Comorbidities are a source of concern and frustration, sometimes eclipsing concern regarding HIV (2) Understanding of health conditions and medications promotes adherence, (3) Simpler regimens with fewer side effects promote adherence, and (4) Untreated substance abuse and mental health issues hinder adherence.

**Conclusions:**

HIV-positive patients in this study voiced concern regarding medical comorbidities and highlighted patient understanding, regimen factors, and substance abuse/mental health issues as barriers to adherence. Addressing these issues may improve outcomes in the aging HIV-positive population. Adherence to medications among HIV-positive patients with DM or HTN may be influenced by providing targeted disease-specific education, simplifying regimens, and treatment of substance abuse/mental health issues.

## Background

Since the widespread adoption of potent antiretroviral therapy (ART) in the United States, AIDS deaths have decreased and HIV has become a chronic illness [1]. As a result, HIV-positive patients have increasing morbidity and mortality from other chronic conditions including diabetes mellitus (DM), hypertension (HTN), and cardiovascular disease [1,2]. DM and HTN are common in HIV-positive patients, with reported DM prevalence in HIV ranging from 2-14% [3,4] and HTN prevalence reported at 31.7% (56.4% in HIV-infected men older than 50) [5]. The prevalence of DM and HTN increases with increasing age. As a result, these conditions will be increasingly important in the management of HIV disease as more people over age 50 are newly infected with HIV and the current HIV-infected population ages [6].

Among HIV-positive patients, comorbidity is common with an average of four to five comorbidities among people older than 50 [7]. Increasing comorbidities are associated with an increase in polypharmacy (taking more than five medications) [7], which may worsen medication adherence due to increased regimen complexity or “pill fatigue”. It is unclear if and how patients take medications for their HIV infection compared to their other comorbid conditions, although early work has shown less adherence to medications for diabetes compared with antiretrovirals [8].

The majority of HIV-positive patients who are engaged in care take ART and achieve an undetectable HIV RNA level [9]. This requires adherence to both clinic visits and medication therapy. Control of other comorbidities requires additional lifestyle changes, such as healthy diet and regular exercise, in addition to adherence to medication therapy. However, little is known about patient perspectives on living with HIV and other chronic comorbidities such as DM and HTN. How HIV-positive patients approach the management of their multiple medical conditions requires further exploration to optimize care of these patients. The objective of this study was to explore HIV-positive patients’ perspectives on living with DM or HTN and factors affecting medication adherence among individuals with HIV and other comorbidities. We hypothesized that among patients with HIV and other comorbidities, adherence to HIV medications and avoiding complications of HIV disease would be of higher priority.

## Methods

### Study design

We conducted six focus groups with a total of 35 participants with HIV and DM or HTN recruited from an HIV clinic in Maryland. All groups were conducted between February and March 2011. We chose focus groups so that the group exchange would facilitate discussion about barriers to and facilitators of treatment adherence when living with multiple medical comorbidities. This study received approval from the Johns Hopkins Medicine Institutional Review Board and all participants provided written informed consent. Participants were ensured of the confidentiality of the group and instructed not to discuss the group proceedings with others.

### Participants

We recruited participants from the clinic using self-referral from flyers and through referral from medical providers. We used this selection process to increase feasibility of recruitment. Potential participants directly telephoned one of the investigators (AM) for a final determination of eligibility for the study. Individuals were eligible to participate if they received HIV care at a Baltimore-area HIV clinic, were on medication therapy for both HIV infection and either DM and/or HTN, were older than 18 and were English-speaking. Medical chart review confirmed the presence of HIV and DM or HTN and medication use. All participants were compensated $50 and two bus tokens for participation in the study.

### Data collection

One of the investigators (TR) led the groups using a discussion guide (Additional file 1) designed to collect data on disease-specific knowledge and attitudes, management of the patients’ various conditions, and barriers to and facilitators of adherence. Groups lasted about one hour. Each group was facilitated by the same investigator (TR), and another investigator (AM) audio-recorded, observed, and took handwritten notes during each group. One investigator (AM) practiced in the clinic from which the participants were recruited, but did not directly care for any of the study participants.

Prior to the group, participants completed a brief questionnaire that included demographic data and data regarding duration of HIV infection and of duration of relationship with care provider. Data collection ceased when thematic saturation occurred.

### Data analysis

Digital recordings of the focus group sessions were transcribed verbatim. Participant identifiers were not transcribed. Two investigators (AM and TR) coded all passages using an editing style analysis, segmenting the data by identifying information pertinent to the research question then categorizing and giving identifying names (codes) to the patterns that emerged [10]. We generated code lists and conceptual themes through an iterative process of discussion and review. This process began with the two investigators separately coding several initial transcripts and then meeting to discuss common findings and key themes. From there, we developed a code book to guide the analysis of the remaining transcripts. Throughout the process, investigators continually met and reviewed findings. Any discrepancies were discussed and resolved by consensus. ATLAS.ti 6.0 (Scientific Software Development GmbH, Berlin) was used for data management and to facilitate analysis. Questionnaire data was analyzed using Stata version 11.0 (College Station, TX).

## Results

### Participant characteristics

The characteristics of study participants (N = 35) are shown in Table 1. The mean age of the overall sample was 51 years (range 43–63), 54% of participants were male, and 91.4% were black. Median duration of relationship with HIV care provider was five years. The median duration of HIV infection was longer than that of DM or HTN.

**Table 1:** Overall sample characteristics (N = 35)

**Characteristic**	**N (%)**
Male sex	19 (54)
Mean age (SD), years	50.8 (5.1)
Black race	33 (94)
HIV/DM	3 (8.6)
HIV/HTN	23 (65.7)
HIV/DM/HTN	9 (25.7)
Median duration of HIV (IQR), years	13 (5, 18)
Median duration of diabetes (IQR), years†	5 (2, 9)
Median duration of hypertension (IQR), years‡	7 (2, 17)
Median duration of relationship with care provider (IQR), years	5 (3, 9)

†Diabetics N = 12.

‡Hypertensives N = 32.

### Factors affecting treatment adherence among people living with HIV and diabetes (DM) and/or hypertension (HTN)

We identified four themes related to medication adherence among patients with HIV and DM or HTN. We present patients’ reflections by theme. Table 2 lists the themes and subthemes, and the number of focus groups in which each theme and subtheme was discussed.

**Table 2:** Themes and subthemes

**Theme**	**Number of groups**
**Theme 1: Comorbidities generate concern and frustration**	**5**
**Theme 2: Understanding of health conditions and medications promotes adherence**	**6**
Subthemes:	
● Understanding the effects of missed doses of medication (6)	6
● Linking disease manifestations and/or symptoms to not taking medication	6
**Theme 3: Simpler regimens with fewer side effects promote adherence**	**4**
**Theme 4: Untreated substance abuse and mental health issues hinder adherence**	**6**
Subthemes:	
● Substance abuse leads to difficulty engaging with and staying in care	5
● Depression contributes to non-adherence to medications and lifestyle recommendations	5

### Theme 1: Comorbidities are a source of concern and frustration, sometimes eclipsing concern regarding HIV

Participants expressed concern about their comorbidities. Achieving control of DM or HTN was perceived as necessary to avoid complications of comorbidities. Participants described family members’ experiences with complications of DM and HTN, including heart attack, dialysis, and limb amputation. Concern about adverse consequences of uncontrolled HTN is illustrated by the following quotations: *“I got the HIV, I ain’t going to worry about that. I have to worry about having a heart attack or a stroke,”* and “*Do I want to live to see my four-year-old grandson, or do I not want to take my high blood pressure medicine and watch my diet and watch what I eat because, like [my doctor] said, HIV is not going to kill me. It’s the high blood pressure.*”

Many participants described that taking their HIV medications had led to suppression of HIV and improvements in health. However, control of comorbidities seemed more elusive, and non-control was a source of frustration, particularly when multiple medications did not control the condition: *“Cause they got me taking like three or four pills for my pressure and it still is not controlling it. So if they could give me like one pill that I could take and it controlled my pressure I’d be better with that,”* and *“My problem for me is my diabetes . . . they just uncontrollable . . . I don’t care what I eat or what I do, they will do what they want to do.”*

In addition to concern about DM and HTN, participants mentioned hepatitis C, asthma/COPD osteoporosis, and sarcoidosis as other comorbid conditions of concern to them. The patients who mentioned conditions with significant physical manifestations (asthma manifested through shortness of breath) reported taking medications for those conditions regularly. This was similar to patients who had physical manifestations of their HIV/AIDS reporting increased adherence to ART (discussed in Theme 2).

### Theme 2: Understanding of health condition reinforces adherence

Patients described the effect of understanding their condition on medication adherence. One subtheme was understanding the consequences of missed doses of medication. Regarding HIV, this was expressed by patients who knew that missed antiretroviral doses lead to resistance, *“And the thing is with Atripla is you can’t miss . . . if you miss, your [drug] levels will go back down. So they’d have to take you off of them and put you on something else.”* Other patients gave less weight to missed doses, which may have reflected less understanding of how the medications work. Alternatively, those patients may not have experienced or may not have been educated about the effects non-adherence. This was expressed both with regards to antiretrovirals, *“I mean, you’ve got it in your system. It’s not like if you miss one day all of those going to be completely gone out of you . . . If you miss a day it ain’t gonna hurt nothing. You just jump back on it tomorrow,”* and blood pressure medications, *“The medicine is . . . cumulative, so you miss a day you shouldn’t get a headache right away . . . because it accumulates in your body.”*

Another subtheme was linking disease manifestations and/or symptoms to not taking medication. Some participants expressed that experiencing physical manifestations of their illnesses motivated them to adhere to prescribed treatments. For example, participants who had been admitted with complications of AIDS or told that they were going to die if they did not take antiretroviral therapy expressed that as a motivating factor for continuing their HIV medications, as follows, *“And she retested me and found out that I was HIV positive . . . I was almost gone when she got me, and she worked with me and said . . . 'You’ve got to do this.’ . . . And it was a no-brainer to me. If I don’t take these medications, I’m gonna die. . . I did everything she told me to do and I’m undetectable.”* Similarly, participants who had experienced physical manifestations of their elevated blood pressure expressed strong motivation to continue with their antihypertensive medications, *“In the morning I take my high blood pressure medicine . . . I know to take it . . . because I don’t want them headaches. I definitely take that every day. And I take mine as prescribed.”* When participants did not feel symptoms of their comorbid illness, less regular adherence was reported, *“With the high blood pressure medicine . . . some days I feel better I don’t take it. I don’t take it like I should . . . it gives [other patients] migraines and things like that. With something like that, if I was at that stage then I would be more sensible of taking it. . . But since I’m not at the stage . . . I take it when I feel . . . that I need it.”*

### Theme 3: Simpler regimens with fewer side effects promote adherence

Participants frequently commented on the burden of their medication regimens. As an extreme example, *“It’s difficult for me because I take 22 pills a day and I take six shots of insulin. That’s just too much medication. . . It’s too much and, you know, I cry every day because I just don’t feel like taking all those pills.”* Furthermore, participants expressed concerns about the side effects of all the different medications, particularly when combined, *“Have you ever watched the TV and they tell you about a simple drug and they then say, okay, but it can cause heart disease, stroke . . . all this just from one pill. So just imagine. You might have 50 side effects from one pill so just imagine all of the side effects you have from the different HIV medicines and your blood pressure medicines going together, ”* and *“One time they put me on two or three different high blood pressure medicines, then I started feeling sleepy and heavy, . . . and the numbers dropped . . . but I was feeling like I was medicated. So then I--well and now I just give up.”*

Patients who did have simplified regimens expressed their satisfaction with those regimens for HIV, *“But this last [regimen], I can thank God that I’m not detectable and . . . this one is great-just one pill once a day,”* and for diabetes, *“my insulin . . . I was taking like 4 shots . . . Now I got the pen. The new pen that I stick myself twice, one in the morning, one in the evening. And that’s awesome.”*

### Theme 4: Untreated substance abuse and mental health issues hinder adherence

Some participants described difficulty engaging in care for HIV and their other conditions because of substance abuse, *“[When] I was getting high smoking crack and using drugs, I really didn’t care whether I was HIV-positive and high blood pressure or anything,”* and, *“Now when I was out there using I wasn’t a responsible addict where I could say well, I’m going to take my HIV medications and blood pressure pills every day.”* They recognized that drug abstinence facilitates medication adherence, as expressed by several participants: *“A lot of people who think that when they hear HIV they think it’s a death sentence . . . but if you start applying to the medication then you’ll see the difference in your life. If you stop getting high your lifestyle will change and start praying or whatever you have to do,”* and, *“[My husband] relapsed and I had to go. Because I know if I relapse, that’s like killing myself. I relapse, I drink a beer, I’m going to the next thing . . . I’m not going to take my medicine. I’m not going to take my medicine or my viral load everything is going, blood pressure, everything. My psych medicine. I’m going to start acting crazy erratic . . . And I have abused my medication before. Getting high. Taking Trazodone to bring me down. Taken Depakote to bring me up. Along with street drugs. I’ve done this, you know.”*

Participants discussed how mental health issues, particularly depression, contributed to non-adherence to medications. *“I got so much depressed where I have missed like, I’d say like three months [of pills], and I had to go back . . . in a psych ward 'cause I was thinking about hurting myself . . . I stayed up there on a psych ward for like a whole month- -'cause I wasn’t ready to come back out on the street.”* Some patients discussed the negative effects of depression on trying to maintain lifestyle modification recommendations for DM or HTN, “*Sometimes my depression takes me to indulge in things that ain’t good for me . . . I had to reflect on that and snap out of it. You know, because before I catch myself, I’m . . . at 280 [pounds].*”

In addition to the themes described above, another factor influencing adherence among some participants was social support. Examples of social support that reinforced adherence were having someone who lived with a participant reminding him or her to take medication and networking with other HIV-positive individuals within and outside of the clinic. A barrier to adherence reported by some participants was navigating the complex systems of insurance and medication coverage, although this was not uniformly reported.

## Discussion

Patients described a variety of factors influencing adherence to their medications for HIV, HTN, and DM. Four main themes emerged: (1) Comorbidities generate concern and frustration, sometimes eclipsing concern regarding HIV (2) Understanding of health conditions and medications promotes adherence, (3) Simpler regimens with fewer side effects promote adherence, and (4) Untreated substance abuse and mental health issues hinder adherence.

It appears that patients’ experiences with HIV, DM, and HTN shaped their perceptions of the importance of the illnesses. They described concerns regarding the adverse consequences of uncontrolled DM and HTN, incorporating information from their experiences with family members and from advice given by their medical providers. Now that it is possible to control HIV with ART, most patients actively engaged in HIV care are able to achieve and maintain an undetectable HIV RNA [9]. Current estimates are that 75 – 79% of individuals maintain an undetectable HIV RNA [11,12], with characteristics of the patient, regimen, clinical setting, and patient/provider relationship potentially limiting adherence [13]. However, when HIV suppression is achieved, patients and providers are able to shift their focus to addressing other chronic conditions like DM and HTN. However, unlike HIV, these other conditions may not be controlled through medication therapy alone. They often entail additional self-care activities (shopping for healthy food, exercising, etc.) that require specific knowledge and skills that can be very time-intensive [14]. In attempting to balance multiple health-related and non-health-related competing demands, patients may not be able to devote the time and resources necessary to control their comorbid conditions. Many participants in our groups, particularly those who had well-controlled HIV, expressed frustration that their DM or HTN was difficult to control despite trying to follow their provider’s recommendations.

Factors contributing to non-adherence include poor understanding of both a medication regimen and of the relationship between antiretroviral non-adherence and antiretroviral resistance [15-17]. Most of our participants described the importance of taking their medications as prescribed. However, those who had experienced HIV-related illness or physical manifestations of DM or HTN mentioned those episodes as particularly strong motivators of adherence.

Better medication adherence has been seen among individuals previously diagnosed with an AIDS-defining opportunistic infection [18] or with more advanced HIV [19] in earlier studies. However, more recent studies either did not examine AIDS-defining diagnosis as an HIV medication adherence predictor [20] or did not find an association [21]. Among hypertensive patients, the belief that variations in blood pressure are symptomatic is related to both better adherence with antihypertensive agents and blood pressure control [22]. However, many chronic conditions are asymptomatic, and treatment of these chronic conditions is primarily to prevent long-term morbidity and mortality, not for short-term quality of life improvement [23] or symptom relief. For patients who have not had symptomatic AIDS or experienced symptoms from their HTN or DM, providers must emphasize the importance of treatment for long-term risk reduction even in the absence of symptoms. Patients act on their beliefs and adhere to medications that help them meet their goals [24]. One potential approach [25] is for providers to align the patient’s beliefs about the identity (symptoms), causes, timeline, control, and consequences of their illness with medical knowledge. In this way, patients will develop an adaptive understanding of their illness, allowing for more productive self-management.

Additional factors reported as affecting adherence were regimen pill burden/regimen complexity and presence of side effects. For some of our focus group participants, the burden of non-antiretroviral regimens was higher than the burden of antiretroviral regimens. Krentz and colleagues [26] attempted to quantify the burden of non-antiretroviral therapy in people with HIV, examining patterns of antiretroviral and non-antiretroviral use among HIV clinic patients over time (1990–2010). They found that after peaking in 1998 at a mean of 12.1 pills per day, the mean number of pills per day in 2010 was 6.7, with 22% of all patients taking more than 10 pills per day, and antiretrovirals accounting for 40-50% of total pill burden. In that study, the highest pill burden was among older patients with longer duration of HIV. As patients with HIV age and have additional comorbidities, the number of daily medications they use will increase, which poses a challenge to continued adherence to all medications.

Finally, participants described how untreated mental health and substance abuse issues affected their medication adherence, both to antiretroviral and non-antiretroviral medications. Depression has consistently been associated with medication nonadherence, not following advice from providers, and missing appointments [27,28]. Thus, identifying and treating depression is a crucial first step in controlling HIV, which is dependent on medication adherence, and controlling other comorbidities, which depends not only on medication adherence but also on developing a therapeutic alliance and instituting lifestyle changes. Substance abuse has also been associated with antiretroviral nonadherence in several large prospective studies in HIV-positive patients [29,30]. Our findings suggest that patients were not adherent to any of their medications when their substance abuse was not controlled, emphasizing the need for effective substance abuse treatment for improving overall health outcomes.

Our data adds to the limited data regarding treatment adherence in patients with HIV and DM or HTN. In a recent study by Batchelder and colleagues of patients with both HIV and DM [8], patients reported better adherence to their antiretroviral medications compared with their DM medications, which was not reported by our study participants. Another finding from the Batchelder study was that DM symptom burden was associated with non-adherence to DM medications. Similarly, our participants described increased HTN-related symptoms when they did not adhere to their antihypertensive medications. Additional findings from the Batchelder study, specifically that concerns regarding antiretroviral side effects and presence of depression were negatively associated with antiretroviral medication adherence, were similar to our results. The authors did not find an association between concerns regarding DM medication side effects or presence of depression with DM medication adherence. They did not collect information regarding substance abuse.

The major limitation of our study is that there is limited generalizability. Qualitative research collects detailed information from a small group, and therefore our findings may not be applicable on a larger scale. The sample was drawn from a single clinic and the participants were racially homogenous (almost entirely African-American). Although more input from members of other racial groups may have provided different insights, the burden of HIV in the United States is disproportionately borne by African-Americans, who represent 14% of the U.S. population but 46% of people living with HIV [31]. African-Americans also have higher rates of DM and HTN, and develop HTN earlier in life [32,33]. Therefore, exploring the perspectives of African-Americans facing HIV and other chronic comorbidities is particularly relevant to the design of future interventions targeting these issues. The median duration of the relationship with the care provider among study participants was five years. Establishment of a strong long-term relationship with a care provider fosters good adherence [34], and our results may have been different if the participants had a shorter relationship duration with their providers.

The major strength of our study is that it is an examination of the intersection of HIV management and comorbidity management. As patients with HIV live longer and experience chronic comorbidities in addition to HIV, these other comorbidities may contribute more to morbidity and mortality than HIV itself, but control is of other comorbidities is often elusive. This research adds to the literature by bringing patients’ voices to the table, which will aid in future intervention design to optimize comorbidity management among individuals with HIV.

## Conclusions

To optimize treatment outcomes among an aging HIV-positive population, control of comorbid DM and HTN is required. Our findings that patient understanding, regimen factors, and substance abuse/mental health issues influence medication adherence are consistent with prior studies regarding treatment adherence in HIV-positive patients. Our results suggest that there multiple targets for improving medication adherence in a population with a high risk for adverse outcomes. Adherence to medications among HIV-positive patients with DM or HTN may be improved by providing targeted disease-specific education, simplifying regimens, and addressing substance abuse/mental health issues.

## Abbreviations

ART: Antiretroviral therapy; DM: Diabetes mellitus; HTN: Hypertension; HIV: Human immunodeficiency virus; HIV RNA: Human immunodeficiency virus ribonucleic acid “HIV viral load”; COPD: Chronic obstructive pulmonary disease; AIDS: Acquired immune deficiency syndrome.

## Competing interests

The authors have no competing interests to disclose.

## Authors’ contributions

AKM conceived of the study; TLR, RDM and GC helped design the study. AKM, TLR, and GC acquired, analysed and interpreted the data. AM drafted the manuscript, TR, RM and GC have revised it critically for important intellectual content. All authors have given final approval of the version to be published.

## Pre-publication history

The pre-publication history for this paper can be accessed here:

http://www.biomedcentral.com/1472-6963/13/488/prepub

## Supplementary Material

Additional file 1 Interview Guide. Click here for file
