# Supplementary material for: Medication adherence in HIV-positive patients with diabetes or hypertension: a focus group study
Source: BMC Health Serv Res. 2013 Nov 25;13:488. doi: 10.1186/1472-6963-13-488 (PMC3907021; doi:10.1186/1472-6963-13-488)
Supplement: Additional file 1 — Interview Guide. [file 1472-6963-13-488-S1.docx]

**Additional material**

**Medication adherence in HIV-positive patients with diabetes or hypertension: A focus group study**

Interview Guide

Question 1

When you think about the terms “treatment adherence,” what comes to your mind?

Question 2

What makes it easy to stick with your medications? What makes it hard to stick with your medications?

Question 3

Has your doctor talked with you about adherence to your medications? Have you ever been enrolled in a program to improve treatment adherence? If yes, tell me about this/these experiences.

Question 4

Which has higher priority for your health, HIV or diabetes/hypertension? Which condition can you control the best?

Question 5

How well has your doctor explained HIV disease? How well has your doctor explained diabetes/hypertension?

Question 6

What suggestions do you have to improve medication taking in people with HIV and diabetes/hypertension?
